# Punicic Acid Triggers Ferroptotic Cell Death in Carcinoma Cells

**DOI:** 10.3390/nu13082751

**Published:** 2021-08-10

**Authors:** Perrine Vermonden, Matthias Vancoppenolle, Emeline Dierge, Eric Mignolet, Géraldine Cuvelier, Bernard Knoops, Melissa Page, Cathy Debier, Olivier Feron, Yvan Larondelle

**Affiliations:** 1 Louvain Institute of Biomolecular Science and Technology (LIBST), UCLouvain, Croix du Sud 4-5/L7.07.03, B-1348 Louvain-la-Neuve, Belgium; perrine.vermonden@uclouvain.be (P.V.); matthias.vancoppenolle@uclouvain.be (M.V.); emeline.dierge@uclouvain.be (E.D.); eric.mignolet@uclouvain.be (E.M.); geraldine.cuvelier@uclouvain.be (G.C.); bernard.knoops@uclouvain.be (B.K.); melissa.page@uclouvain.be (M.P.); cathy.debier@uclouvain.be (C.D.); 2 Pole of Pharmacology and Therapeutics (FATH), Institut de Recherche Expérimentale et Clinique (IREC), UCLouvain, 57 Avenue Hippocrate B1.57.04, B-1200 Brussels, Belgium; olivier.feron@uclouvain.be

**Keywords:** conjugated linolenic, acids, punicic acid, carcinoma cells, ferroptosis, docosahexaenoic acid, spheroids, lipid peroxidation

## Abstract

Plant-derived conjugated linolenic acids (CLnA) have been widely studied for their preventive and therapeutic properties against diverse diseases such as cancer. In particular, punicic acid (PunA), a conjugated linolenic acid isomer (C18:3 c9t11c13) present at up to 83% in pomegranate seed oil, has been shown to exert anti-cancer effects, although the mechanism behind its cytotoxicity remains unclear. Ferroptosis, a cell death triggered by an overwhelming accumulation of lipid peroxides, has recently arisen as a potential mechanism underlying CLnA cytotoxicity. In the present study, we show that PunA is highly cytotoxic to HCT-116 colorectal and FaDu hypopharyngeal carcinoma cells grown either in monolayers or as three-dimensional spheroids. Moreover, our data indicate that PunA triggers ferroptosis in carcinoma cells. It induces significant lipid peroxidation and its effects are prevented by the addition of ferroptosis inhibitors. A combination with docosahexaenoic acid (DHA), a known polyunsaturated fatty acid with anticancer properties, synergistically increases PunA cytotoxicity. Our findings highlight the potential of using PunA as a ferroptosis-sensitizing phytochemical for the prevention and treatment of cancer.

## 1. Introduction

For centuries, plant-derived lipids have been used for their therapeutic properties against various diseases [1,2]. Fatty acids are the building blocks of natural lipids and represent a large class of compounds that are diverse in composition. They act as a source of energy as well as structural and functional components of cells [1]. Saturated fatty acids have no double bond while unsaturated fatty acids present at least one double bond in their carbon chain [3]. Polyunsaturated fatty acids (PUFAs) are classified according to the number and position of double bonds in the carbon chain. Most PUFAs present double bonds separated by a methylene (-CH_2_-) group. In contrast, some PUFAs display double bonds that are not interrupted by a methylene group and are known as conjugated fatty acids [1,4]. Conjugated linoleic acids (CLAs) have two conjugated double bonds while conjugated linolenic acids (CLnAs) have three double bonds of which at least two are conjugated [5]. The three double bonds of plant-derived CLnAs are in a conjugated configuration. In contrast to CLAs that are particularly found in dairy products, CLnAs are mainly contained in diverse plant seed oils, such as pomegranate seed oil rich in punicic acid (PunA, C18:3 c9t11c13) and tung, bitter gourd or ricinodendron seed oil rich in α-eleostearic acid (α-ESA, C18:3 c9t11t13) [6,7,8].

Among the conjugated fatty acids, CLAs have been the most extensively studied for their beneficial effects on human health. These include anti-obesity, anti-atherogenic, anti-diabetic, anti-carcinogenic and immunomodulatory properties [7,9,10,11,12,13]. However, the interest towards CLnAs has significantly increased over the last two decades, partially due to their high content in some seed oils, suggesting that CLnAs may be more available for preventive or even therapeutic purposes than previously expected [2]. In fact, CLnAs have been shown to possess anti-inflammatory [14,15,16], anti-obesity [14,17,18], anti-diabetic [19,20] and anti-cancer activities [1,2,21,22,23]. Specifically, PunA has been reported to exert a strong anti-cancer activity, both in vitro [7,24,25,26] and in vivo [4,27,28]. PunA anti-cancer activity is thought to be associated with lipid peroxidation [7]. In fact, CLnAs are more susceptible to autoxidation than their non-conjugated counterpart, namely α-linolenic acid (C18:3 c9c12c15), due to the ease of free radical formation by the quick electron delocalization at the level of the conjugated double bonds [29]. However, the exact mechanisms behind PunA cytotoxicity towards cancer cells remain poorly understood. 

Ferroptosis is a form of iron-catalyzed regulated cell death that is morphologically, biochemically and genetically distinct from other regulated cell deaths, such as apoptosis and necroptosis [30,31]. Ferroptotic cell death is characterized by the overwhelming accumulation of lipid hydroperoxides, a form of reactive oxygen species (ROS) generated through oxidation of PUFAs [32,33,34,35]. This mode of cell death is executed through PUFA-containing phospholipid peroxidation as well as the presence of redox-active iron and a defective lipid peroxide repair (i.e., deficiency in glutathione and impairment of glutathione peroxidase 4) [36]. More recently, other key ferroptosis suppressors have been identified, such as the ferroptosis suppressor protein 1, which regenerates the radical trapping-reduced form of ubiquinone and the Ca^2+^-independent phospholipase A_2_β, which hydrolyses lipid hydroperoxides from cell membranes [37,38]. Mechanistically, the formation of lipid hydroperoxides requires di-oxygenation of lipid double bonds, which occurs either spontaneously by autoxidation or through enzyme-catalyzed processes controlled by lipoxygenases [39,40,41] and oxidases [42]. Accumulation of lipid hydroperoxides eventually leads to the disruption of cell membranes, production of reactive aldehydes and finally cell death.

In the last decade, multiple drugs impacting the activity of various enzymes or transporters have been identified as ferroptosis inducers in cancer cells [30,43,44,45]. Another strategy, as yet largely unexplored for the induction of ferroptosis, may lie in the promotion of lipid hydroperoxide production by preferentially introducing high amounts of peroxidable PUFAs in cancer cells. In fact, several lines of research suggest that a wide range of PUFAs might sensitize cancer cells to ferroptosis by causing a dramatic accumulation of phospholipid-derived peroxides [37,46,47,48]. A very recent study conducted by Beatty et al. showed that upon incorporation into triglycerides, α-ESA triggers ferroptosis in triple negative breast cancer cells [49]. This study however is at odds with previous work from our team, where we reported that triglyceride storage has a protective effect against PUFA-induced ferroptosis [50]. More work is thus warranted to further dissect the potential pro-ferroptotic effects of PunA. This is even more necessary as PunA, unlike other CLnAs, is a readily available phytochemical. It is indeed present in large amounts in pomegranate seed oil [6], the only CLnA-rich oil widely recognized as edible on the market [14]. While tung oil intake (as used in the study of Beatty et al. [49]) has been associated with toxic effects in vivo [51], the consumption of pomegranate seed oil has demonstrated no detrimental effects on body functions nor on tissue homeostasis [17,52].

In the present work, we show that PunA is cytotoxic for hypopharyngeal and colorectal carcinoma cells in vitro, either grown in monolayers or as three-dimensional spheroids. Our data indicate that PunA induces ferroptosis in carcinoma cells by triggering an intense lipid peroxidation, a phenomenon that is prevented by ferroptosis inhibitors. In addition, a combination of PunA with docosahexaenoic acid (DHA, C22:6 c4c7c10c13c16c19), another PUFA recently shown to induce ferroptosis under acidosis [50], increases its effect in a synergistic manner. These findings suggest that PunA, possibly in combination with DHA, could be used as an anti-cancer agent.

## 2. Materials and Methods

### 2.1. Cell Culture

Human colorectal HCT-116 and hypopharyngeal FaDu cancer cell lines were purchased from ATCC (CCL-247 and HTB-43). Cell lines were stored in liquid nitrogen in DMEM supplemented with 10% heat-inactivated FBS (Sigma, Saint-Louis, MO, USA) and 5% DMSO (Sigma, Saint-Louis, MO, USA). All cells were cultured in DMEM supplemented with 10% heat-inactivated FBS, 10 mM D-glucose, 2 mM L-glutamine and 25 mM of PIPES and HEPES and adjusted to pH 7.4 with NaOH 5 M (Merck, Darmstadt, Germany). All cell lines tested negative for mycoplasma contamination using the MycoAlert^TM^ Mycoplasma Detection kit (Lonza, Basel, Switzerland).

### 2.2. Cell Viability Assessment

Before being tested, fatty acids were conjugated to bovine serum albumin (BSA, Sigma, Saint-Louis, MO, USA) in phosphate buffer saline to obtain a fatty acid/BSA ratio of 4:1 (*w*/*w*). Cells were seeded at a density of 10,000 cells/well in 96-well tissue culture treated plates (Greiner Bio One, Frickenhausen, Germany, 655180) and incubated at 37 °C, 5% CO_2_. After 24 h, cell culture medium was supplemented with fixed concentrations of BSA-conjugated punicic acid (Larodan, Solna, Sweden, 10-1875) or docosahexaenoic acid (Larodan, Solna, Sweden, 10-2206), as well as of (+)-α-tocopherol (Sigma, Saint-Louis, MO, USA), ferrostatin-1 (Sigma, Saint-Louis, MO, USA), deferoxamine mesylate (Sigma, Saint-Louis, MO, USA), ZVAD-fmk (Sigma, Saint-Louis, MO, USA) or necrostatin-1 (Sigma, Saint-Louis, MO, USA) for 72 h. All compounds were dissolved in DMSO, except (+)-α-tocopherol, which was dissolved in ethanol. The doses of punicic acid were chosen to cover a large range of concentrations while the doses of the other compounds were selected based on previous studies [39,41,50]. After 72 h, cell viability was determined by using Presto Blue reagent (Thermo Fisher Scientific, Waltham, MA, USA) according to manufacturer’s instructions. In brief, viable cells are metabolically active and, therefore, are able to reduce the blue non-fluorescent dye (rezasurin) into a pink colored fluorescent molecule (resorufin).

### 2.3. Fatty Acid Quantification

Fatty acid quantification was performed both on preparations of fatty acids conjugated to BSA, in order to precisely determine their concentrations, and on cell culture media that were collected 18 h post-treatment and used to determine the fatty acid uptake by carcinoma cells, in parallel with the quantification of lipid peroxidation-derived products (see below). Firstly, total lipids were extracted with methanol:chloroform:water (2:2:1; *v:v:v*) with the Bligh and Dyer technique [53]. An internal standard composed of nonadecanoic acid (Larodan, Solna, Sweden, 10-1900-16) was added in each sample to evaluate the extraction yields. Samples were dried under a stream of nitrogen at 30 °C. Secondly, dried lipids were methylated under alkaline conditions (0.5 mL of KOH 0.1 M in methanol at 70 °C for 1 h) and further under acidic conditions (addition of 0.2 mL of HCl 1.2M in methanol at 70 °C for 15 min). Fatty acid methyl esters (FAMEs) were extracted with 1 mL of hexane. Thirdly, methyl-undecanoate (Larodan, Solna, Sweden, 20-1100-13) was added in each sample as an injection standard. FAMEs were injected and separated by gas chromatography as previously described [8]. An external standard composed of the combination of 43 pure methyl ester standards (Larodan and Nu-Check Prep) was used to identify the unknown peaks with the retention time and to quantify the peaks through the known concentrations. A punicic acid methyl ester standard of known concentration (Larodan, Solna, Sweden, 20-1875) was used to identify and quantify the punicic acid peak in each sample. Chromatograms were processed using ChromQuest 5.0 software (Thermo Fisher Scientific, Waltham, MA, USA). Results are expressed as the ratio of the final amount to the initial amount of each fatty acid added in the cell culture media, after data normalization with the internal standard (percentage of recovery), the injection standard and the starting cell number. 

### 2.4. 3-D Spheroid Models

Spheroids were formed with HCT-116 or FaDu cells seeded at a density of 1000 cells/well in 96-well ultra-low attachment plates (Greiner Bio One, Frickenhausen, Germany, 650970) in DMEM supplemented with 10% heat-inactivated FBS, 10 mM D-glucose, 2 mM L-glutamine and 25 mM of PIPES and HEPES and adjusted to pH 7.4. Three days after seeding (corresponding to day 0), spheroids were treated with 14 µM of punicic acid or with 100 µM of docosahexaenoic acid, 0.2 or 10 µM of (+)-α-tocopherol or ferrostatin-1, either alone or in combination with 14 µM of punicic acid. The treatment was renewed every 2 or 3 days. Spheroid growth was evaluated using a live-cell microscope equipped with a microscope camera (Moticam 3.0 MP) and the spheroid diameter was measured based on the magnifying power and the ratio between the picture and the microscope field of view. All spheroid samples from a same experiment were imaged with the same exposure and gain settings.

### 2.5. Measurement of Lipid Peroxidation Potential (BODIPY Assay)

Cells were seeded in a 96-well black/clear bottom plates (Greiner Bio One, Frickenhausen, Germany, 655096). Peroxidation potential was assessed using a fluorescent lipid probe C_11_-BODIPY^581/591^(4,4-difluoro-5-(4-phenyl-1,3-butadienyl)-4bora-3a,4a-diaza-s-indacene-3 undecanoic acid; Thermo Fisher Scientific, Waltham, MA, USA) comprising 2 double bonds, which are oxidized in the presence of ROS. Upon oxidation, the probe color changes from non-peroxidized (red fluorescence) to peroxidized (green fluorescence). Cells were incubated with 5 µM of C_11_-BODIPY^581/591^ for 30 min at 37 °C. Then, they were washed with PBS and the red and green fluorescence levels were evaluated using a Spectramax iD3 microplate reader (Molecular Devices) (red: λ_excitation_ = 580 nm, λ_emission_ = 620 nm; green: λ_excitation_ = 500 nm and λ_emission_ = 540 nm). The wavelengths were selected after optimization and spectral range detection. Data are expressed as relative fluorescence units determined by dividing the green fluorescence by the red fluorescence and calculating the ratio between the value obtained for the test condition and the one obtained for the control condition.

### 2.6. Quantification of Lipid Peroxidation-Derived Products (MDA Assay)

Malondialdehyde (MDA) concentration in cellular extracts was assessed with the Lipid peroxidation (MDA) assay kit from Merck (Darmstadt, Germany, MAK085) according to manufacturer’s instructions.

### 2.7. Statistics

Statistical analyses were performed with JMP 14 and GraphPad Prism 8.4 software by using two-way ANOVA with Sidak’s multiple comparison tests. Statistical significance to the control or to another treatment is determined as follows: * *p* ≤ 0.05/(*n* − 1); ** *p* ≤ 0.01/(*n* − 1); *** *p* ≤ 0.001/(*n* − 1), with *n* being the number of different compared conditions.

## 3. Results

### 3.1. PunA Is Cytotoxic to Different Carcinoma Cell Lines

**Figure 1:**
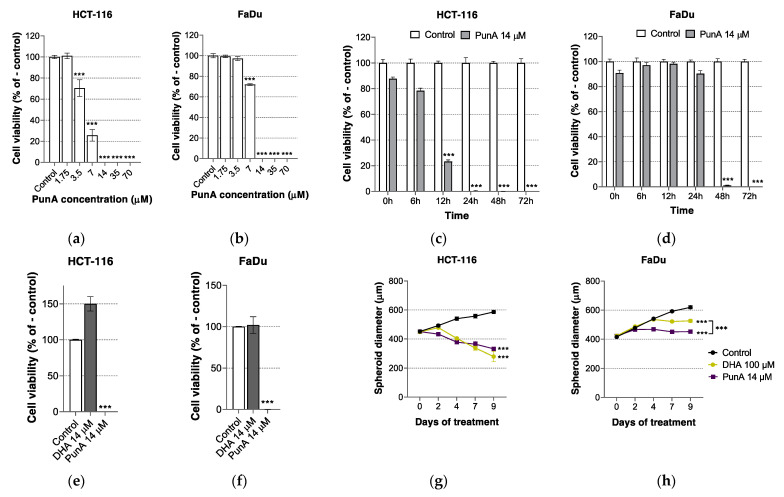
Punicic acid (PunA) decreases the viability of HCT-116 and FaDu carcinoma cells and its effect arises from a lower dose as compared to docosahexaenoic acid (DHA). (**a**) Cytotoxic effect of increasing concentrations of PunA on HCT-116 and (**b**) FaDu carcinoma cells after 72 h; (**c**) Kinetics of the cytotoxicity of PunA 14 µM over 72 h on HCT-116 and (**d**) FaDu carcinoma cells; (**e**) Comparison of the cytotoxicity of PunA and DHA at 14 µM on HCT-116 and (**f**) FaDu carcinoma cells applied during 72 h; (**g**) Comparison of the diameter growth of HCT-116 or (**h**) FaDu 3D spheroids under PunA 14 µM or DHA 100 µM. Control: cells or spheroids supplemented with DMEM cell culture medium without added fatty acid. Results are expressed as mean ± standard error of the mean of three independent repetitions. Significance is established in relation to the control (**a**,**b**,**e**–**h**), to PunA 14 µM at 0 h of treatment (**c**,**d**) or between DHA 100 µM and PunA 14 µM (**g**–**h**). *** *p* ≤ 0.0001.

PunA is cytotoxic to HCT-116 and FaDu carcinoma cells (Figure 1a,b). With a dose of 7 µM of PunA, both HCT-116 and FaDu carcinoma cells faced a significant cytotoxic effect with 25% and 70% remaining viability after 72 h, respectively (inhibitory concentration IC50 of 5 and 9 µM). At a dose above 10 µM, PunA led to the complete death of both cell types after 72 h. With regard to the kinetics of PunA cytotoxicity, HCT-116 carcinoma cells appear to be more sensitive to PunA cytotoxicity than FaDu cells (Figure 1c,d). Indeed, the viability of HCT-116 cells significantly decreased after 12 h of treatment whereas FaDu cell viability was only impacted after more than 24 h of treatment, suggesting that PunA sensitivity depends on the cell line origin.

The cytotoxicity of DHA on carcinoma cells has been shown in the past [54,55,56,57] and has been recently demonstrated on carcinoma cells under acidosis [50]. Here we show that at micromolar doses, PunA but not DHA decreases the viability of carcinoma cells grown in physiological conditions (Figure 1e,f). At a dose of 14 µM during 72 h, only PunA was cytotoxic to carcinoma cells, with a dramatic loss of viability in both HCT-116 and FaDu cells, whereas DHA had no cytotoxic impact and even increased HCT-116 cell viability. Of note, DHA showed no or only a limited cytotoxicity at pH 7.4, even at 100 µM (80% (HCT-116) and 100% (FaDu) viability after 72 h, data not shown). Regarding the carcinoma cells grown as 3D spheroids, PunA cytotoxicity arose from a lower dose as compared to DHA, with a 7-fold lower dose of PunA leading to a similar (HCT-116) or stronger (FaDu) decrease in spheroid diameter as compared to DHA (Figure 1g,h). Of note, DHA 100 µM alone led to a reduction in spheroid diameter as compared to the control, likely due to its cytotoxicity on carcinoma cells facing acidosis inside the spheroids [50]. Again, HCT-116 carcinoma cells appear more sensitive to PunA than FaDu cells both in time and in cytotoxic response, with a lower final diameter for HCT-116 spheroids. Interestingly, the cytotoxic effect of PunA appears to be less strong on 3D spheroids than on carcinoma cells cultured in monolayers for both cell lines. Indeed, despite repeated treatment over time, PunA only induced a partial cell detachment from 3D spheroids (Supplementary Materials File Figure S1), suggesting that cell death did not occur evenly throughout the spheroid. Such a phenomenon may be explained by stronger cell-to-cell interactions in 3D spheroids creating a dense barrier, which may limit or delay the migration of fatty acids to the inner part of the spheroids.

### 3.2. The Effect of PunA on Carcinoma Cells Is Enhanced in the Presence of DHA

**Figure 2:**
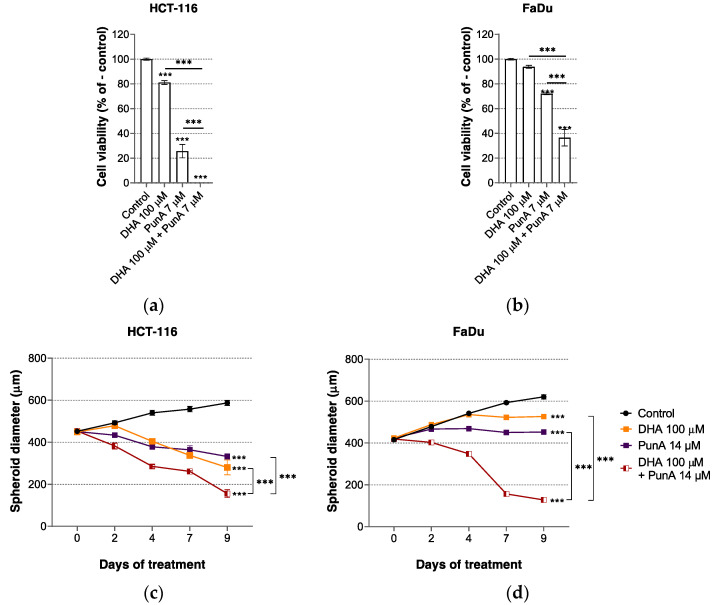
Combining punicic acid (PunA) with docosahexaenoic acid (DHA) increases its cytotoxicity on HCT-116 and FaDu carcinoma cells. (**a**) Viability of HCT-116 and (**b**) FaDu carcinoma cells treated with DHA, PunA or combination thereof. (**c**) Diameter growth of HCT-116 and (**d**) FaDu 3D spheroids treated with PunA 14 µM, DHA 100 µM or combination thereof. Control: cells or spheroids supplemented with DMEM cell culture medium without added fatty acid(s). Results are expressed as mean ± standard error of the mean of three independent repetitions. Significance is established in relation to the control or between DHA or PunA alone and their combination. *** *p* ≤ 0.0001.

The distinct cytotoxic profile of DHA and PunA led us to examine the effects of their combination on carcinoma cells. We found that the viability of HCT-116 and FaDu carcinoma cells exposed to both DHA at 100 µM and PunA at a sub-lethal dose of 7 µM for 72 h was more greatly reduced than upon treatment with PunA alone (Figure 2a,b). The combination of PunA with DHA also significantly decreased spheroid growth (vs. single fatty acid treatments) (Figure 2c,d). These findings suggest that PunA and DHA act in a supra-additive way to impact on carcinoma cell viability and that their combination may thus possess an interesting anti-cancer potential to be exploited in preventive or therapeutic strategies.

### 3.3. PunA Effect Is Inhibited by Ferroptosis Inhibitors

**Figure 3:**
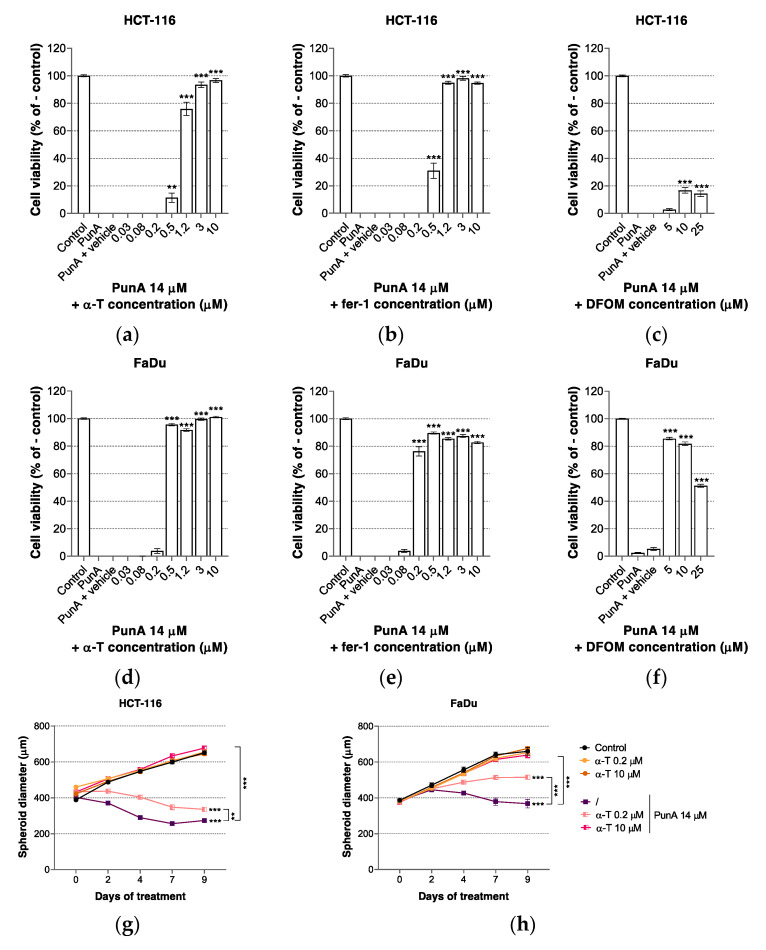

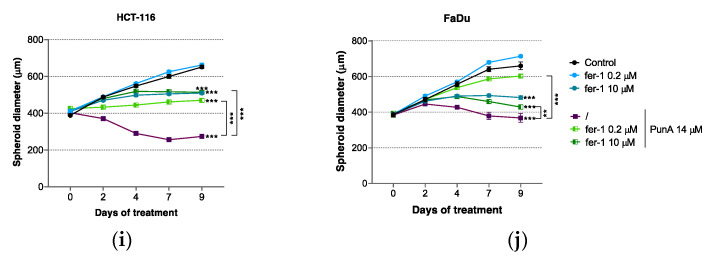
The effect of punicic acid (PunA) is inhibited by the addition of ferroptosis inhibitors. (**a**–**e**) Viability of HCT-116 carcinoma cells in the presence of PunA 14 µM and an increasing dose of (**a**) α-tocopherol (α-T), (**b**) ferrostatin-1 (fer-1) or (**c**) deferoxamine mesylate (DFOM). (**d**,**f**) Viability of FaDu carcinoma cells in the presence of PunA 14 µM and an increasing dose of (**d**) α-T, (**e**) fer-1 or (**f**) DFOM. (**g**,**h**) Diameter growth of (**g**) HCT-116 and (**h**) FaDu 3D spheroids treated with PunA 14 µM and/or 0.2 or 10 µM of α-T. (**i**,**j**) Diameter growth of (**i**) HCT-116 and (**j**) FaDu 3D spheroids treated with PunA 14 µM and/or 0.2 or 10 µM of fer-1. Control: cells supplemented with DMEM cell culture medium without added fatty acid or spheroids supplemented with DMEM cell culture medium containing 0.3% *v/v* of DMSO. Vehicle: ethanol absolute for α-T (0.4% *v/v*) or DMSO for fer-1 and DFOM (0.4% *v/v*). Results are expressed as mean ± standard error of the mean of three independent repetitions. Significance is established in relation to the PunA + vehicle condition (**a**–**f**), to the control (**g**–**j**) or to PunA 14 µM alone (**g**–**j**). ** *p* ≤ 0.00143; *** *p* ≤ 0.0001.

CLnAs have been reported to have a particularly high rate of peroxyl radical generation due to the triene structure of their double bonds [58,59]. Since ferroptosis relies on an extensive accumulation of PUFA-derived lipid peroxides [32,33], we assumed that PunA may trigger ferroptosis in carcinoma cells. Although some previous reports indicated that CLnAs are likely to trigger apoptosis [7,23,60], ferroptosis has recently appeared as a potent cell death pathway underlying CLnA cytotoxicity on cancer cells [49]. In the present study, we evaluated the impact of two ferroptosis inhibitors, namely ferrostatin-1 (fer-1) and α-tocopherol (α-T), on the cytotoxicity of PunA on HCT-116 and FaDu carcinoma cells. Upon insertion in cell membranes, fer-1 and α-T have been reported to largely slow down the accumulation of lipid hydroperoxides, thereby preventing ferroptosis. While α-T is widely used as a natural lipophilic antioxidant, fer-1 is a more potent and specific xenobiotic low molecular weight inhibitor of ferroptosis [30,61]. PunA cytotoxicity on HCT-116 and FaDu carcinoma cells was inhibited by the addition of fer-1 and α-T, as well as by the iron chelator deferoxamine mesylate (DFOM) (Figure 3a–f). These results are consistent with previous reports indicating that both lipophilic antioxidants and iron chelators inhibit lipid hydroperoxide generation during ferroptosis [43,62]. On the contrary, neither the apoptosis inhibitor ZVAD-fmk nor the necroptosis inhibitor necrostatin-1 prevented PunA cytotoxicity on HCT-116 and FaDu carcinoma cells, further supporting ferroptosis as the cell death pathway triggered upon PunA exposure (Supplementary Material File Figure S2). None of the inhibitors were cytotoxic for HCT-116 and FaDu carcinoma cells when applied alone (Supplementary Materials File Figure S3a–d,f–i). In contrast, both HCT-116 and FaDu cancer cells appear to be sensitive to iron chelation since DFOM alone induced a significant loss of viability at doses above 25 µM (Supplementary Material File Figure S2e,j). A reason for such a sensitivity to iron chelation may be related to the reliance of cancer cells on various iron-dependent enzymes or pathways [63]. Interestingly, the alleviation of PunA cytotoxicity on HCT-116 carcinoma cells by DFOM reached approximately 20% while FaDu carcinoma cells recovered more than 80% viability, showing again a difference in cell line sensitivity to PunA cytotoxicity.

Both α-T and fer-1 inhibited the cytotoxicity of PunA on HCT-116 and FaDu spheroids (Figure 3g–j). A concentration of α-T as low as 0.2 µM was sufficient to significantly mitigate the inhibitory effects of PunA on spheroid growth while at 10 µM, α-T completely prevented the cytotoxicity of PunA on HCT-116 and FaDu spheroids after 9 days of treatment (Figure 3g,h). In both HCT-116 and FaDu spheroids, PunA induced a broad cell detachment over the treatment period, an event that was completely prevented by α-T addition (Supplementary Material File Figure S4). Fer-1 similarly reduced PunA cytotoxicity on 3D spheroids (Figure 3i,j). However, fer-1 as a single agent also decreased HCT-116 and FaDu spheroid growth after repeated treatments at a dose of 10 µM. Consistent with the inhibitory effect on spheroid diameter growth, the cell detachment induced by PunA was prevented by the addition of fer-1 on both HCT-116 and FaDu spheroids (Supplementary Material File Figure S4). These results converge toward ferroptosis as a mechanism that underlies PunA cytotoxicity on carcinoma cells.

### 3.4. PunA Treatment Leads to an Increase in Intracellular Lipid Peroxidation

**Figure 4:**
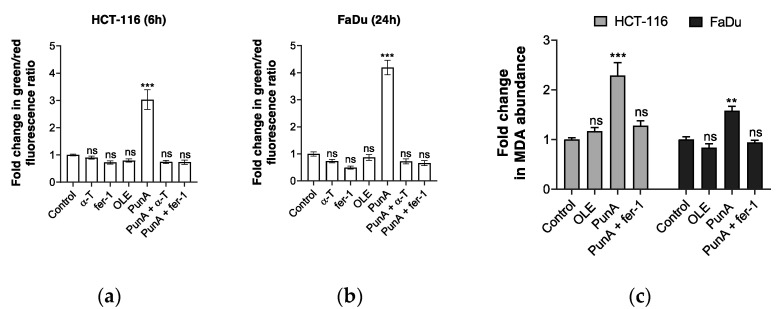
Punicic acid (PunA) treatment largely increases lipid peroxidation in carcinoma cells. (**a**,**b**) Fold change in the BODIPY green/red fluorescence ratio compared to the control for (**a**) HCT-116 and (**b**) FaDu carcinoma cells treated with α-tocopherol (α-T) 10 µM, ferrostatin-1 (fer-1) 10 µM, oleic acid (OLE) 50 µM, PunA 14 µM, PunA 14 µM + α-T 10 µM or PunA 14 µM + fer-1 10 µM. HCT-116 were treated for 6 h while FaDu cells were treated for 24 h based on kinetic results. (**c**) Fold change in MDA abundance (nmol MDA/mg protein) compared to the control for HCT-116 and FaDu carcinoma cells treated with OLE 50 µM, PunA 14 µM or PunA 14 µM + fer-1 10 µM. HCT-116 and FaDu carcinoma cells were treated for 18 and 36 h, respectively, based on their viability kinetics. Control: cells supplemented with DMEM cell culture medium without added fatty acid(s). Results are expressed as mean ± standard error of the mean of three independent repetitions. Significance is established in relation to the control. ns, non-significant; ** *p* ≤ 0.001; *** *p* ≤ 0.0001.

As ferroptosis execution is characterized by an abundant accumulation of lipid peroxide species [35], we investigated whether PunA treatment triggers an increase in lipid peroxidation in carcinoma cells. We used the C11-BODIPY assay [47,48], which measures the ability of cells to peroxidize the double bonds of this BODIPY probe. We showed that PunA treatment induced a 3- and 4.2-fold rise in lipid peroxidation in HCT-116 and FaDu carcinoma cells after 6 and 24 h, respectively (Figure 4a,b); these effects of PunA were prevented in the presence of either α-T or fer-1 10 µM (Figure 4a,b). Of note, oleic acid did not trigger intracellular lipid peroxidation (Figure 4a,b), which is not surprising since that monounsaturated fatty acid has rather been reported to reduce the sensitivity of membrane lipids to peroxidation [64]. The treatment time after which the BODIPY fluorescence was measured is based on the kinetics of PunA cytotoxicity and the difference in sensitivity between the two cell lines (see Figure 1c,d). Of note, no fold change in lipid peroxidation was observed after 6 h in FaDu carcinoma cells and lipid peroxidation could not be measured after 24 h of treatment in HCT-116 due to a complete loss of cell viability (data not shown). As the BODIPY assay correlates with the potential of the cells to initiate lipid peroxidation [62], the data indicate that PunA increased the ability of both HCT-116 and FaDu carcinoma cells to initiate the generation of peroxyl radicals.

The MDA assay measures the secondary products resulting from intracellular lipid peroxidation [65]. After 18 and 36 h of PunA treatment, HCT-116 and FaDu carcinoma cells showed a 2.4- and 1.6-fold increase in MDA abundance as compared to untreated cells, respectively, indicating a significant intracellular lipid peroxidation (Figure 4c). Consistent with ferroptosis, fer-1 inhibited PunA-induced increase in MDA abundance while oleic acid did not trigger any significant change in MDA abundance as compared to control cells (Figure 4c). In addition, the rise in MDA abundance after PunA treatment correlates with fatty acid uptake since PunA disappeared from both HCT-116 and FaDu cell culture media to a similar extent after 18 and 36 h, respectively (Table 1). As the MDA assay quantifies the adducts between lipid peroxidation-derived aldehydes and the thio-barbituric acid reagent, lipid peroxidation must already be at an advanced stage to allow measuring a significant rise in MDA abundance. HCT-116 and FaDu carcinoma cells showed a significantly reduced viability after 18 and 36 h, respectively (see Figure 1c,d), suggesting that lipid peroxidation may have occurred sufficiently enough to detect its derived products. Therefore, these timings were selected to measure the MDA abundance upon PunA exposure. Consistently, FaDu carcinoma cells did not show any rise in MDA abundance after 18 h of PunA treatment (Supplementary Material File Figure S5). Taken together, these findings indicate that PunA triggers lipid peroxidation in carcinoma cells in a manner and a time course that are consistent with the induction of ferroptosis.

## 4. Discussion

Despite great advances in treatment, cancer remains the second cause of mortality worldwide, with the majority of cancer deaths caused by carcinoma [66]. Many anti-cancer drugs aim at triggering apoptosis as a strategy to eliminate cancer cells. However, the effectiveness of these drugs is limited by the tendency of cancer cells to acquire resistance to apoptosis [67]. In this regard, exploiting other types of cell death mechanisms such as ferroptosis opens up new therapeutic avenues. PunA, a CLnA isomer, is cytotoxic to different carcinoma cell lines by triggering intracellular lipid peroxidation and these effects are completely prevented in the presence of ferroptosis inhibitors (Figure 3 and Figure 4). Neither apoptosis nor necroptosis inhibitors blocked PunA cytotoxicity, further supporting the ability of PunA to specifically trigger ferroptosis in cancer cells (Supplementary Material File Figure S2). Ferroptotic effects of PunA were observed from low concentrations, both on carcinoma cells grown as monolayers and on cells organized as three-dimensional spheroids.

Our findings support the concept that exploiting highly peroxidable CLnAs to foster the accumulation of lipid hydroperoxides may be a new anti-cancer strategy. Although dihydroxy derivatives of CLnAs have been reported to be responsible for CLnA cytotoxicity [68], further investigations are required to determine whether lipid peroxidation is driven by non-enzymatic autoxidation of PunA incorporated into specific lipid species or is under the control of specific pro-oxidative enzymes. Another point to explore is the intracellular location of accumulated PunA and how PunA incorporation into specific lipid species influences the initiation of ferroptosis. Although PUFA-containing phospholipid residues have been shown to be the prime substrates leading to ferroptosis [47,48], the involvement of other lipid species such as cardiolipins and neutral lipids in CLnA cytotoxicity deserves more investigation. Indeed, the conjugated structure of CLnAs confers them a chemical specificity that lipid enzymes could tackle differently as compared to unconjugated PUFAs. Interestingly, Beatty et al. showed that the incorporation of α-ESA into neutral lipids is responsible for its pro-ferroptotic effect [49]. However, the different CLnA isomers have been documented to be distributed differently between neutral lipids and phospholipids based on their number of trans double bonds, suggesting that the cytotoxicity of specific CLnA isomers may be due to their incorporation into selective lipid species [5]. Interestingly, the tumor microenvironment may also influence PunA cytotoxicity on carcinoma cells, for example via various metabolites produced by immune cells, such as NO released by macrophages [69].

Our results indicate that DHA and PunA may work synergistically to induce cell death in carcinoma cells. To our knowledge, this is the first study suggesting synergies between PUFA and CLnA cytotoxicities on cancer cells and spheroids. Although n-3 PUFAs such as DHA have shown promising anti-cancer effects both in vitro [50,70,71,72] and in vivo [50,55,73], clinical trials that prove their therapeutic exploitation remain limited and fail to provide clear evidence of their anti-cancer effects in patients [3]. Because CLnAs are less studied compared to PUFAs and their efficacy in vivo remains a matter of debate, clinical trials that aim at demonstrating the anti-cancer effects of CLnAs in humans are simply lacking [14]. Even though in vivo evidence of their synergistic mechanism should be provided, such a combination of PUFAs and CLnAs may be an interesting therapeutic option to increase their respective effects in cancer patients.

As indicated by our results as well as by other in vitro and in vivo studies [1,49], CLnAs are phytochemicals with promising anti-cancer effects that could be exploited as preventive or even therapeutic agents. CLnAs are mainly found in seed oils of specific plants and the CLnA content can reach up to 80% of the total lipids [5], making CLnAs accessible nutritional phytochemicals. However, CLnA-rich seed oils remain too little known and available on the market to be used on a large scale as anti-cancer agents. Only pomegranate seed oil is currently widely recognized as an edible oil on the market [14], making PunA the most relevant CLnAs to be further investigated as a potential anti-cancer agent. A strategy to make these phytochemicals more available could be to include CLnAs in frequently consumed food items. Recent studies reported that laying hens fed with pomegranate seed oil significantly incorporate PunA in egg yolks [8,74]. Another study reported that PunA was successfully incorporated in margarine [75] and in goat milk of animals fed with a diet supplemented with 12% of pomegranate seed pulp [76]. A different approach would be the production of enriched food supplements that could be taken up by cancer patients. Paul and colleagues suggested that delivering CLnA isomers in the form of nano-emulsions would enhance CLnA bioavailability and storage stability [77]. However, as an oral intake of PunA would mainly lead to its incorporation into triglycerides as part of complex lipids (i.e., chylomicrons and lipoproteins), whether cancer cells are able to take up PunA in the form of triglycerides should be carefully investigated. Such enriched food products and supplements open up new opportunities to promote the use of CLnAs as health beneficial phytochemicals in the context of cancer prevention and treatment. Further studies are, however, needed to assess whether PunA-induced lipid peroxidation may cause adverse effects on the long term.

## Figures and Tables

**Table 1:** Fatty acid relative absorption by carcinoma cells after 18 or 36 h. Ratio of the final to the initial amount (nmol) of the corresponding fatty acid in cell culture medium in contact with HCT-116 or FaDu carcinoma cells for 18 or 36 h, respectively, upon treatment with oleic acid (OLE) 50 µM, PunA 14 µM or PunA 14 µM + ferrostatin-1 (fer-1) 10 µM. 3 million cells were exposed to 8 mL of cell culture medium supplemented with the corresponding treatment. Results are expressed as mean ± standard error of the mean of three independent repetitions. Significance is established in relation to the initial amount of the corresponding fatty acid in cell culture medium (considered as 100%). *** *p* ≤ 0.0001.

Cell Treatment	HCT-116	FaDu
OLE 50 µM	8.74 ± 2.14 ***	9.02 ± 3.37 ***
PunA 14 µM	7.53 ± 1.21 ***	4.79 ± 1.53 ***
PunA 14 µM + fer-1 10 µM	4.94 ± 0.28 ***	2.66 ± 0.38 ***

## Data Availability

Data sharing is not applicable to this article.

## Supplementary Materials

The following are available online at https://www.mdpi.com/article/10.3390/nu13082751/s1. Figure S1: Morphological characterization of HCT-116 and FaDu 3D spheroids treated with punicic acid (PunA) or docosahexaenoic acid (DHA); Figure S2: Neither the apoptosis inhibitor ZVAD-fmk nor the necroptosis inhibitor necrostatin-1 prevent punicic acid (PunA) cytotoxicity; Figure S3: None of the inhibitors have a cytotoxic effect when applied alone whereas iron chelation impacts on cell viability; Figure S4: Morphological characterization of HCT-116 and FaDu 3D spheroids treated with punicic acid (PunA) and/or ferroptosis inhibitors; Figure S5: Punicic acid (PunA) treatment for 18 h does not increase lipid peroxidation in FaDu carcinoma cells.
