# Supplementary material for: Punicic Acid Triggers Ferroptotic Cell Death in Carcinoma Cells
Source: Nutrients. 2021 Aug 10;13(8):2751. doi: 10.3390/nu13082751 (PMC8399984; doi:10.3390/nu13082751)
Supplement: Supplementary file 1 [file nutrients-13-02751-s001.zip › nutrients-1290314-supplementary.pdf]

SUPPLEMENTARY MATERIALS

Punicic Acid Triggers Ferroptotic Cell Death in Carcinoma Cells

Perrine Vermonden [1], Matthias Vancoppenolle [1], Emeline Dierge [1,2], Eric Mignolet [1], Géraldine Cuvelier [1], Bernard Knoops [1], Melissa Page [1], Cathy Debier [1], Olivier Feron [2,†] and Yvan Larondelle [1,*,†]

[1] Louvain Institute of Biomolecular Science and Technology (LIBST), UCLouvain, Croix du Sud 4-5/L7.07.03, B-1348 Louvain-la-Neuve, Belgium; perrine.vermonden@uclouvain.be (P.V.); matthias.vancoppenolle@uclouvain.be (M.V.); emeline.dierge@uclouvain.be (E.D.); eric.mignolet@uclouvain.be (E.M.); geraldine.cuvelier@uclouvain.be (G.C.); bernard.knoops@uclouvain.be (B.K.); melissa.page@uclouvain.be (M.P.); cathy.debier@uclouvain.be (C.D.)

[2] Pole of Pharmacology and Therapeutics (FATH), Institut de Recherche Expérimentale et Clinique (IREC), UCLouvain, 57 Avenue Hippocrate B1.57.04, B-1200 Brussels, Belgium; olivier.feron@uclouvain.be

* Correspondence: yvan.larondelle@uclouvain.be; Tel.: +32-478449925

† These authors have equally supervised this work.

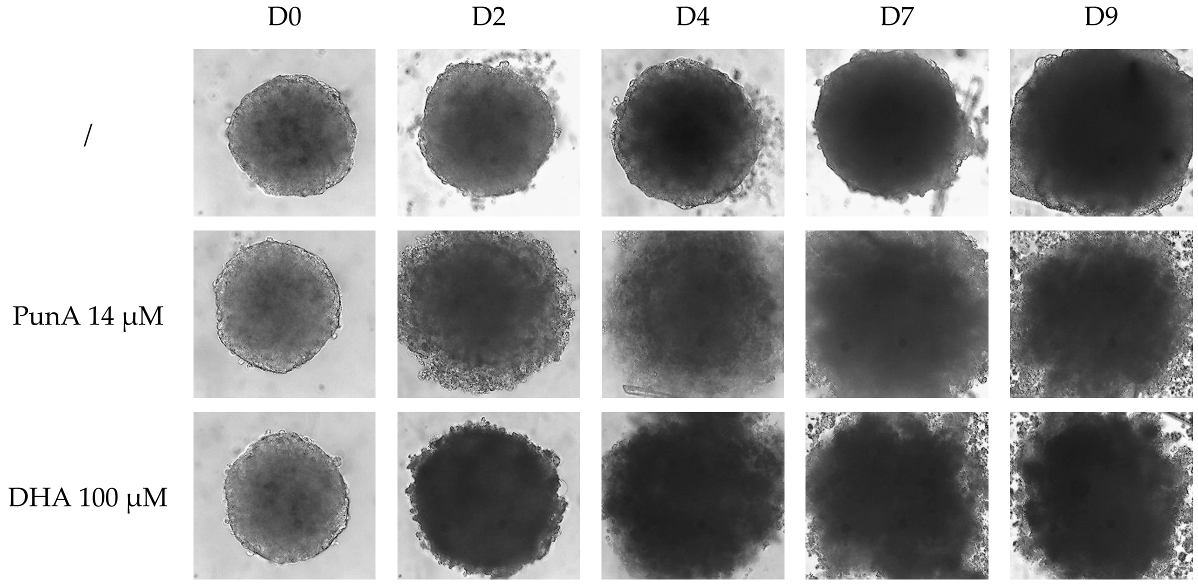

(**a**)

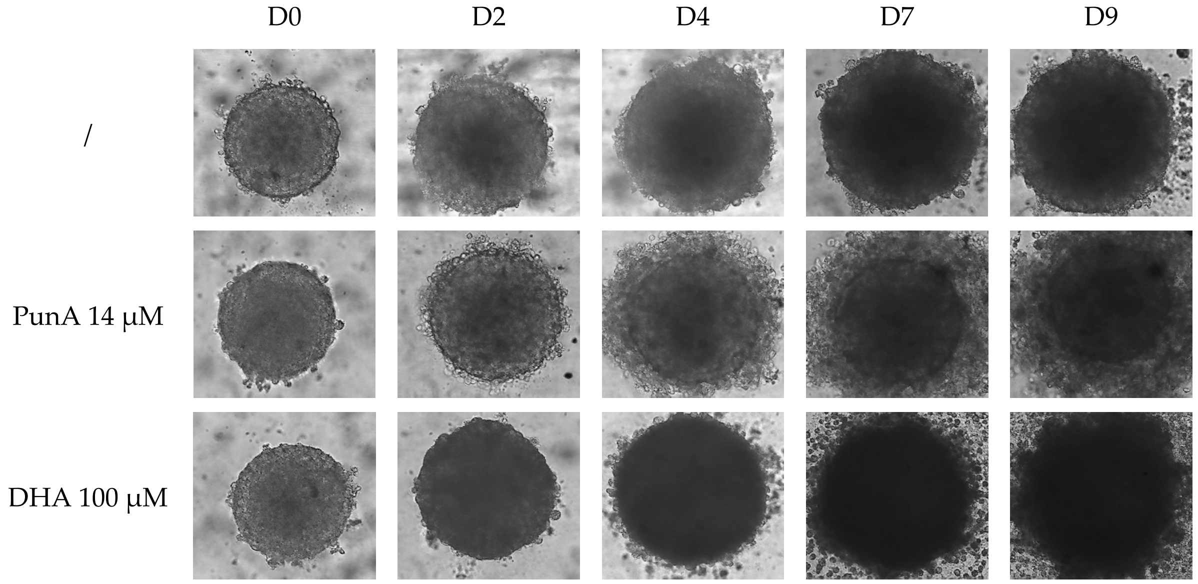

(**b**)

Figure S1. Morphological characterization of HCT-116 and FaDu 3D spheroids treated with punicic acid (PunA) or docosahexaenoic acid (DHA). Representative pictures of (**a**) HCT-116 and (**b**) FaDu 3D spheroids treated with a control, PunA 14 µM or DHA 100 µM over 9 days.

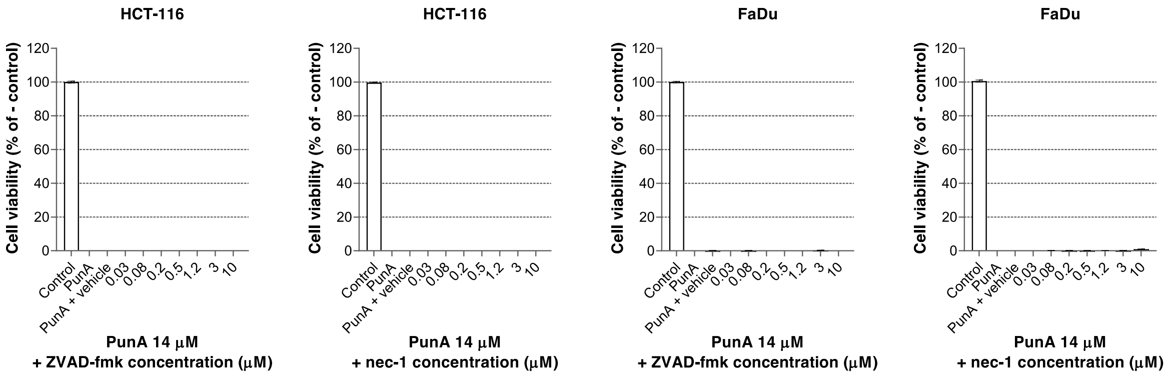

(a) (b) (c) (d)

Figure S2. Neither the apoptosis inhibitor ZVAD-fmk nor the necroptosis inhibitor necrostatin-1 prevent punicic acid (PunA) cytotoxicity. (**a**–**b**) Viability of HCT-116 carcinoma cells in the presence of PunA 14 μM and an increasing dose of (**a**) ZVAD-fmk or (**b**) necrostatin-1 (nec-1). (**c**–**d**) Viability of FaDu carcinoma cells in the presence of PunA 14 μM and an increasing dose of (**c**) ZVAD-fmk or (**d**) necrostatin-1 (nec-1). Control: cells supplemented with DMEM cell culture medium without added fatty acid. Vehicle: DMSO (0.4% *v*/*v*). Results are expressed as mean ± standard error of the mean of three independent repetitions. Significance is established in relation to the PunA + vehicle condition.

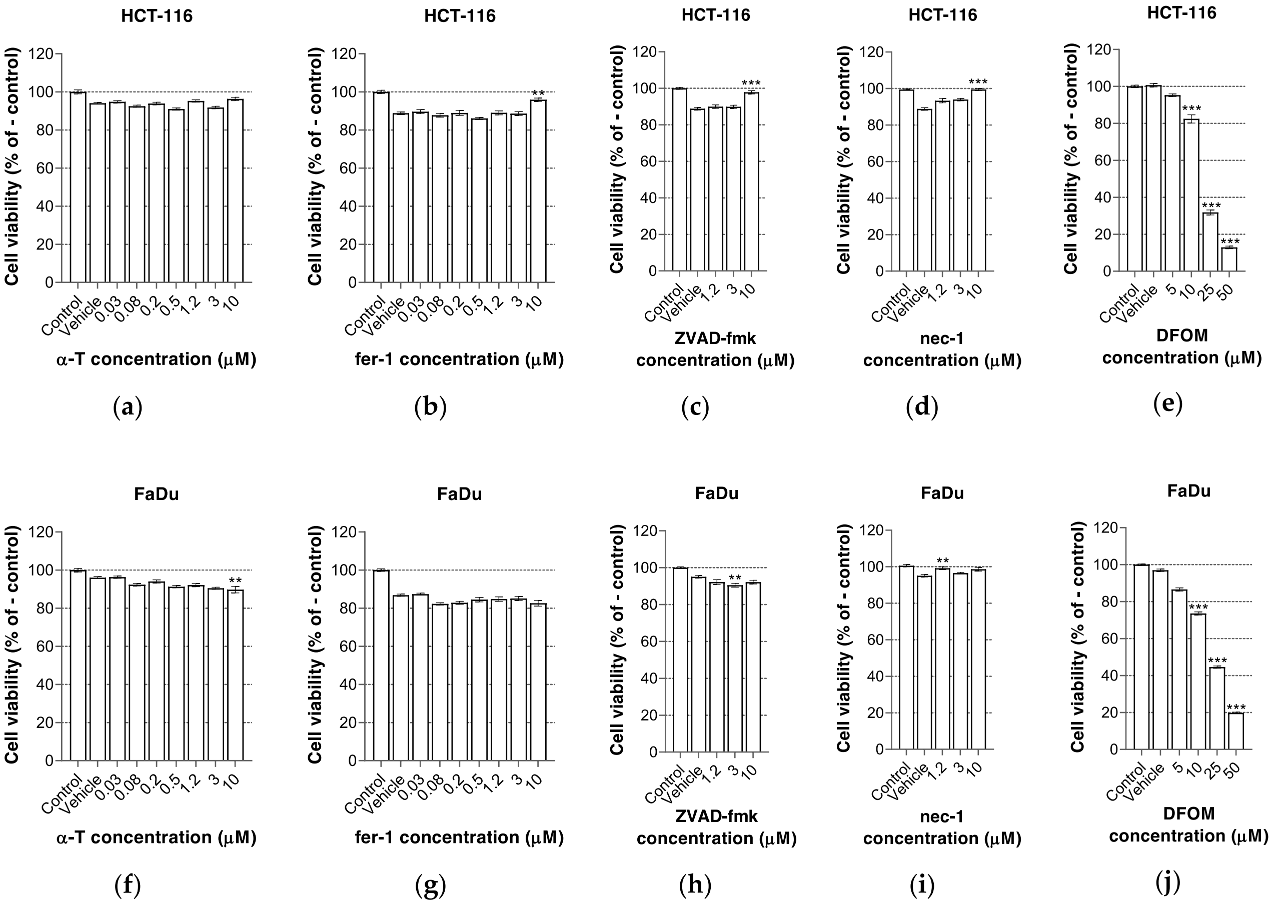

Figure S3. None of the inhibitors have a cytotoxic effect when applied alone whereas iron chelation impacts on cell viability. (**a**–**e**) Viability of HCT-116 carcinoma cells in the presence of an increasing dose of (**a**) α-tocopherol (α-T), (**b**) ferrostatin-1 (fer-1), (**c**) ZVAD-fmk, (**d**) necrostatin-1 (nec-1) or (**e**) deferoxamine mesylate (DFOM). (**f**–**j**) Viability of FaDu carcinoma cells in the presence of an increasing dose of (**a**) α-T, (**b**) fer-1, (**c**) ZVAD-fmk, (**d**) nec-1 or (**e**) DFOM. Control: cells supplemented with DMEM cell culture medium without added compounds. Vehicle: ethanol absolute for α-T (0.4% v/v) or DMSO for fer-1, ZVAD-fmk, nec-1 or DFOM (0.4% *v*/*v*). Results are expressed as mean ± standard error of the mean of three independent repetitions. Significance is established in relation to the vehicle condition. ** $p \leq 0.00125$ (for α-T and fer-1); ** $p \leq 0.0025$ (for ZVAD-fmk and nec-1); *** $p \leq 0.0001$.

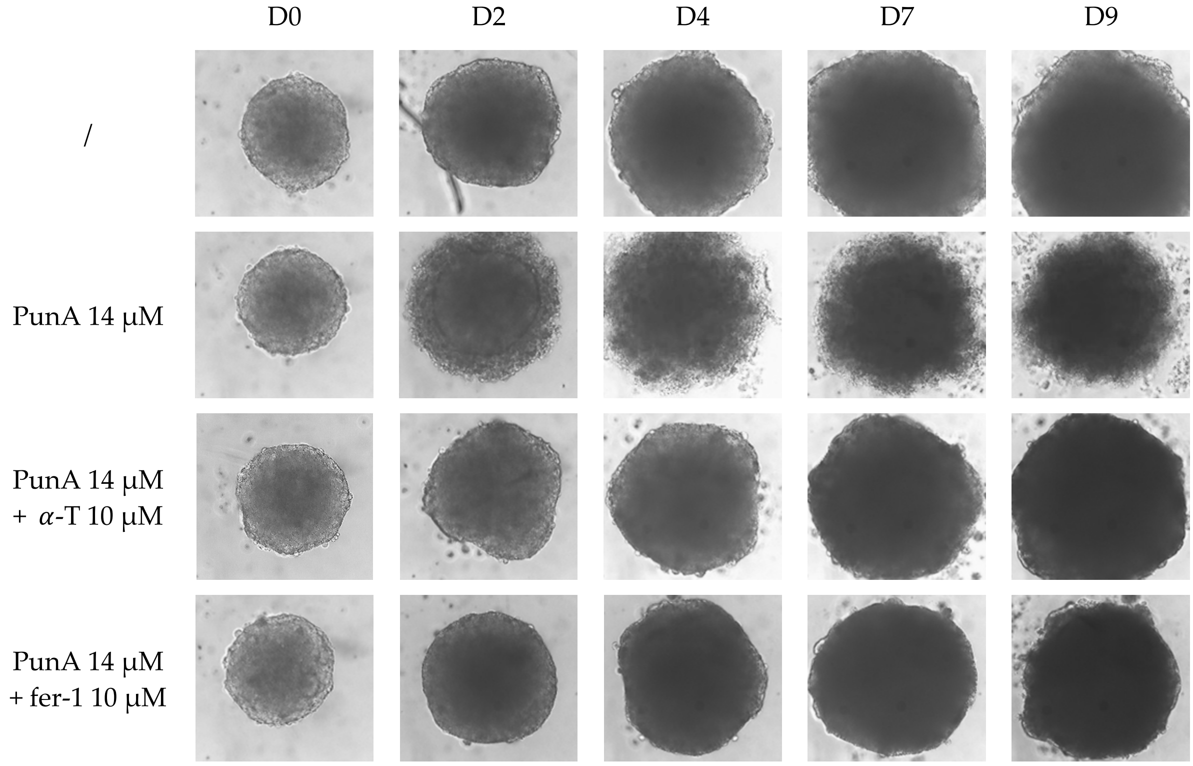

(**a**)

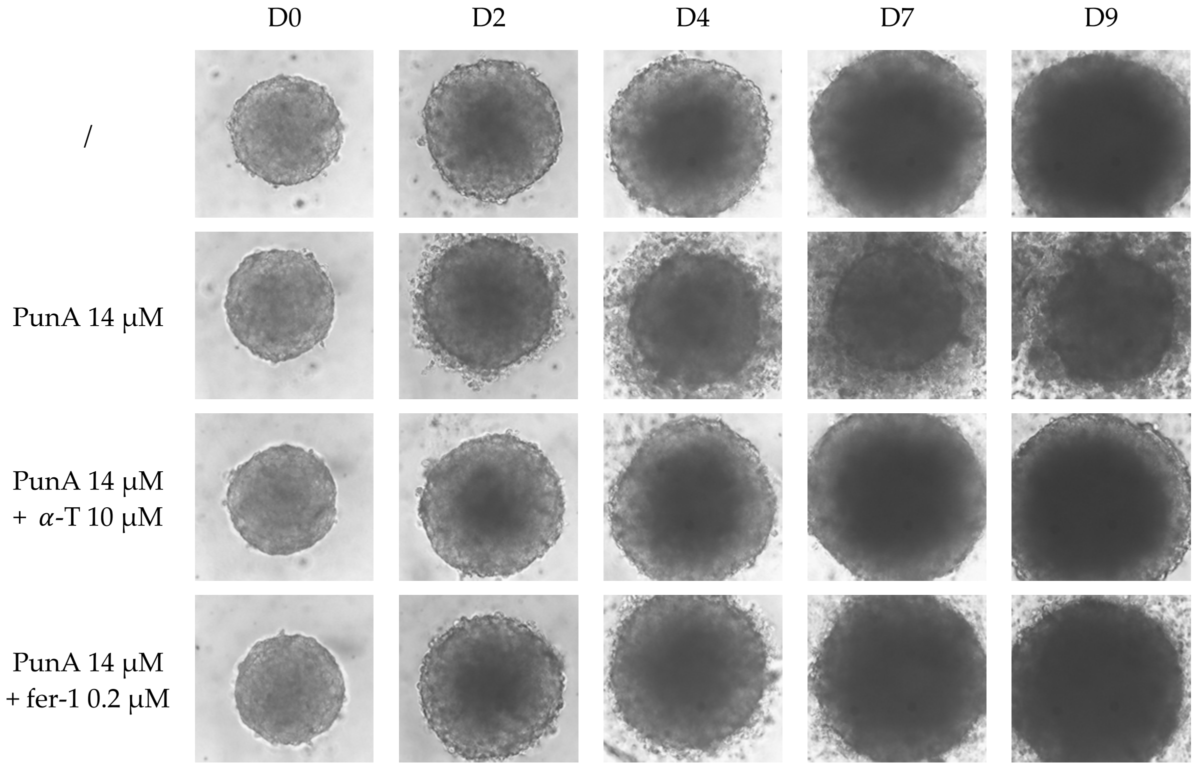

(**b**)

Figure S4. Morphological characterization of HCT-116 and FaDu 3D spheroids treated with punicic acid (PunA) and/or ferroptosis inhibitors. Representative pictures of (**a**) HCT-116 and (**b**) FaDu 3D spheroids treated with a control, PunA 14 μM, PunA 14 μM + α-tocopherol 10 μM (α-T) or PunA 14 μM + ferrostatin-1 (fer-1) 0.2 or 10 μM over 9 days.

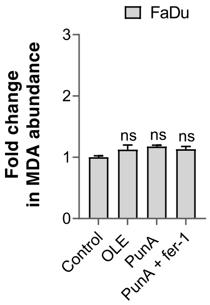

Figure S5. Punicic acid (PunA) treatment for 18 hours does not increase lipid peroxidation in FaDu carcinoma cells. Fold change in MDA abundance (nmol MDA/mg protein) compared to the control for FaDu carcinoma cells treated with OLE 50 μM, PunA 14 μM or PunA 14 μM + fer-1 10 μM for 18 hours. Control: cells supplemented with DMEM cell culture medium. Results are expressed as mean ± SEM of three independent repetitions. Significance is established in relation to the control. ns, non-significant.
